# Effect of Shear Stress on *Pseudomonas aeruginosa* Isolated from the Cystic Fibrosis Lung

**DOI:** 10.1128/mBio.00813-16

**Published:** 2016-08-02

**Authors:** Jozef Dingemans, Pieter Monsieurs, Sung-Huan Yu, Aurélie Crabbé, Konrad U. Förstner, Anne Malfroot, Pierre Cornelis, Rob Van Houdt

**Affiliations:** a Department of Bioengineering Sciences, Research Group Microbiology, Vrije Universiteit Brussel, and VIB Structural Biology, Brussels, Belgium; b Unit of Microbiology, Expert Group Molecular and Cellular Biology, Institute for Environment, Health and Safety, Belgian Nuclear Research Centre (SCK CEN), Belgium; c Institute for Molecular Infection Biology, University of Würzburg, Würzburg, Germany; d Laboratory of Pharmaceutical Microbiology, Ghent University, Ghent, Belgium; e Cystic Fibrosis Clinic and Pediatric Infectious Diseases, Universitair Ziekenhuis Brussel, Vrije Universiteit Brussel (UZB), Brussels, Belgium

## Abstract

Chronic colonization of the lungs by *Pseudomonas aeruginosa* is one of the major causes of morbidity and mortality in cystic fibrosis (CF) patients. To gain insights into the characteristic biofilm phenotype of *P. aeruginosa* in the CF lungs, mimicking the CF lung environment is critical. We previously showed that growth of the non-CF-adapted *P. aeruginosa* PAO1 strain in a rotating wall vessel, a device that simulates the low fluid shear (LS) conditions present in the CF lung, leads to the formation of in-suspension, self-aggregating biofilms. In the present study, we determined the phenotypic and transcriptomic changes associated with the growth of a highly adapted, transmissible *P. aeruginosa* CF strain in artificial sputum medium under LS conditions. Robust self-aggregating biofilms were observed only under LS conditions. Growth under LS conditions resulted in the upregulation of genes involved in stress response, alginate biosynthesis, denitrification, glycine betaine biosynthesis, glycerol metabolism, and cell shape maintenance, while genes involved in phenazine biosynthesis, type VI secretion, and multidrug efflux were downregulated. In addition, a number of small RNAs appeared to be involved in the response to shear stress. Finally, quorum sensing was found to be slightly but significantly affected by shear stress, resulting in higher production of autoinducer molecules during growth under high fluid shear (HS) conditions. In summary, our study revealed a way to modulate the behavior of a highly adapted *P. aeruginosa* CF strain by means of introducing shear stress, driving it from a biofilm lifestyle to a more planktonic lifestyle.

## IMPORTANCE

Biofilm formation by *Pseudomonas aeruginosa* is one of the hallmarks of chronic cystic fibrosis (CF) lung infections. The biofilm matrix protects this bacterium from antibiotics as well as from the immune system. Hence, the prevention or reversion of biofilm formation is believed to have a great impact on treatment of chronic *P. aeruginosa* CF lung infections. In the present study, we showed that it is possible to modulate the behavior of a highly adapted transmissible *P. aeruginosa* CF isolate at both the transcriptomic and phenotypic levels by introducing shear stress in a CF-like environment, driving it from a biofilm to a planktonic lifestyle. Consequently, the results obtained in this study are of great importance with regard to therapeutic applications that introduce shear stress in the lungs of CF patients.

## INTRODUCTION

*Pseudomonas aeruginosa* is a Gram-negative gammaproteobacterium that can dwell in a wide range of environments, including water, soil, animal hosts, and the human host (1). Although this bacterium is harmless to the healthy human host, it poses great danger for individuals that suffer from burn wounds, immunodeficiency, and, in particular, cystic fibrosis (CF) and is therefore considered an opportunistic pathogen (2, 3). This opportunistic lifestyle is facilitated by the multitude of virulence factor-encoding genes present in its large (6-to-7-Mbp) genome (4, 5) as well as by its high metabolic versatility (6). The lungs of CF patients can initially be infected by *P. aeruginosa* via two different routes, namely, by environmental strains that have no clinical case history or by the transmission of adapted strains colonizing other CF patients (7–11). Since the development of whole-genome sequencing approaches (12), more insight into the adaptation of environmental *P. aeruginosa* strains to the CF lung has been gained. Multiple studies have reported that *P. aeruginosa* strains acquire deletions in genes that appear to be less vital for persistence in the CF lung environment, while the horizontal acquisition of novel genomic content has been less frequently observed (13–17). Additionally, *P. aeruginosa* rewires its global regulatory networks in order to survive in the hostile CF lung environment, which is characterized by the presence of immune cells, competing pathogens, and excessive antibiotic treatment (9, 18, 19). Furthermore, it is believed that the shear stress in the CF lung is low due to the presence of viscous sputum that impairs the shear-causing mucociliary movement (20, 21).

Previously, we used rotating wall vessel (RWV) bioreactor technology to study the response of *P. aeruginosa* to low fluid shear (LS) and high fluid shear (HS) regimes (22, 23). The RWV is a cylindrical bioreactor that, when completely filled and rotated on an axis parallel with the ground, results in solid body mass rotation of the culture medium, hence creating a low fluid shear environment (24). Addition of different types of beads or horizontal positioning of the RWV has been reported to enhance fluid shear levels in the RWV (22–24). Low fluid shear conditions were previously shown to affect gene expression and phenotypic traits of the pathogens *Salmonella enterica* serovar Typhimurium, *Escherichia coli*, and *Staphylococcus aureus* compared to those seen with controls under higher fluid shear conditions (24–27).

Using the RWV, we previously demonstrated that culturing of the *P. aeruginosa* PAO1 reference strain in LB medium in the LS environment of the RWV bioreactor leads to the formation of biofilms in suspension (22). However, when the fluid shear in the RWV module was increased by means of a ceramic bead, a phenotype of greater surface attachment of biofilms was observed. Furthermore, the AlgU alternative sigma factor mainly appeared to orchestrate this response to LS conditions, resulting in elevated levels of the exopolysaccharide alginate (23). These data suggested that the RWV bioreactor creates environmental conditions that trigger phenotypic traits in *P. aeruginosa* relevant to the conditions seen in CF lung mucus, since alginate-containing biofilms are among the hallmarks of chronic *P. aeruginosa* infections in this environment (28–30). However, the PAO1 reference strain has no CF background, since it was originally isolated from a wound (31, 32), and LB medium does not mimic the content and viscosity of sputum present in the lung environment of CF patients. Recently, we have reported the presence of a transmissible *P. aeruginosa* CF strain in Belgian CF patients, distributed among different CF reference centers (13). This strain has been found in CF reference centers for more than 10 years, and whole-genome sequencing revealed that its adaptation to the CF lung conditions involved the accumulation of numerous deletions.

In this study, we scrutinized the effect of shear stress on the behavior of this well-characterized, highly adapted, and transmissible CF strain at the transcriptomic, biofilm, and quorum sensing (QS) levels in artificial sputum medium (ASM) using the RWV bioreactor. Biofilm formation in response to shear stress was assessed via scanning electron microscopy (SEM), while an RNA sequencing (RNA-Seq) approach was adopted to determine the effect of shear stress on the transcriptome of *P. aeruginosa* CF_PA39. In addition, small RNAs (sRNAs) in the genome of this strain were *de novo* predicted and the expression of these sRNAs next to previously confirmed sRNAs in other *P. aeruginosa* genomes was quantified. Finally, the production of both short-chain and long-chain (3-oxo-C12-homoserine lactone [HSL]) QS molecules was determined to assess the role of QS in the response of *P. aeruginosa* CF_PA39 to fluid shear.

## RESULTS AND DISCUSSION

### Two different colony morphologies were identified after growth of *P. aeruginosa* CF_PA39 in artificial sputum medium.

**FIG 1:**
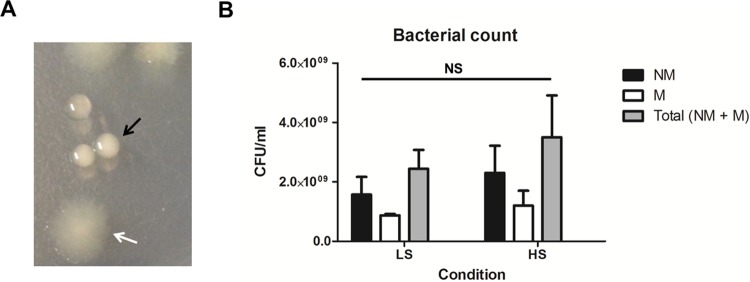
(A) Phenotypes of colonies that were recovered from the RWV bioreactor after 24 h of growth in ASM and subsequently plated on LB medium. Mucoid colonies are indicated by a black arrow, while nonmucoid colonies are indicated by a white arrow. (B) Quantification of bacteria that were recovered from the RWV bioreactor after 24 h of growth in ASM and subsequently plated on LB medium. LS, low fluid shear. HS, high fluid shear. NM, nonmucoid colonies. (M) Mucoid colonies. NS, not statistically significant (*P* > 0.05).

Growth of the CF lung-adapted transmissible *P. aeruginosa* CF_PA39 strain in ASM in the RWV bioreactor resulted in the formation of nonmucoid and mucoid colony morphologies (Fig. 1A). More specifically, nonmucoid colonies were more abundant than mucoid colonies under both low fluid shear (LS) and high fluid shear (HS) conditions, while no statistically significant difference in the numbers of nonmucoid or mucoid colonies was observed in comparisons of the two sets of culture conditions (Fig. 1B). The ratios of nonmucoid to mucoid colonies recovered under LS and HS conditions were 1.78 and 1.92, respectively. Although the overall viable count was slightly higher under HS conditions (3.50 × 10^9^ ± 1.41 × 10^9^ CFU/ml) than under LS conditions (2.44 × 10^9^ ± 6.36 × 10^8^ CFU/ml), the differences between the two sets of conditions were not statistically significant. A similar observation has been reported by Woo and colleagues (33) from a study in which a chronic *P. aeruginosa* CF isolate was inoculated into a flow cell and four extra colony morphotypes were obtained from the biofilm effluent next to the mucoid wild type after 9 days of growth. When the genetic basis of this short-term diversification was revealed, it was found that many of the phenotypic variants from the dispersal population had acquired mutations in genes involved in alginate biosynthesis and c-di-GMP metabolism (34). In order to test whether ASM itself induces the emergence of the nonmucoid phenotype, *P. aeruginosa* CF_PA39 was grown in culture tubes under shaking conditions for up to 124 h. We found that *P. aeruginosa* CF_PA39 switched from the mucoid phenotype (at 24 h of growth) to a solely nonmucoid phenotype (after 124 h of growth) in ASM over time (see Fig. S1 in the supplemental material). In contrast, the majority (ca. 80%) of *P. aeruginosa* CF_PA39 colonies remained mucoid over 124 h of growth in LB medium (see Fig. S2). These results indicate that the ASM medium itself induces *P. aeruginosa* CF_PA39 to diversify. Recently, it was shown that phenotype switching can occur after experimental evolution of *Pseudomonas fluorescens* SBW25 under fluctuating conditions that mimic exposure to the host immune system (35). Although genotypes that were able to switch phenotype acquired nine different mutations, it was revealed that the *casB* gene, involved in the pyrimidine biosynthetic pathway, was responsible for this phenomenon (36). Mutation of this gene led to reduced levels of intermediates in the pyrimidine biosynthetic pathway, hence forcing the cell to make a choice between polymer production (leading to capsulated cells) and nucleotide metabolism (leading to noncapsulated cells). Additionally, it has been shown that growth of *P. fluorescens* SBW25 in a spatially heterogeneous environment leads to the diversification of this strain (37–39). The resulting adaptive radiation led to the emergence of three major phenotypes, each of which occupied a niche with oxygen content different from that of the others. It is likely that steep oxygen gradients formed in the ASM that was used in this study, both under shaking conditions (culture tubes) and under rotating conditions (RWV), due to the viscous properties of this medium. On the other hand, the viscosity of the ASM might select against the energetically costly overproduction of alginate by the mucoid phenotype of the highly adapted *P. aeruginosa* CF_PA39 strain. Nevertheless, it should be mentioned that the behavior of *P. aeruginosa* in the multispecies community of the CF lung could be different, since a number of studies have shown that interspecies competition can reduce intraspecies variation (40, 41). Nonetheless, the occurrence of the nonmucoid phenotype under both LS and HS conditions might have been the result of genetic diversification of a population during growth in ASM under conditions resembling CF lung conditions. The nonmucoid phenotype might represent a more motile dispersal variant that is able to colonize a newly developed niche as a consequence of biofilm development and/or maturation.

### High fluid shear levels preclude the formation of self-aggregating biofilms.

**FIG 2:**
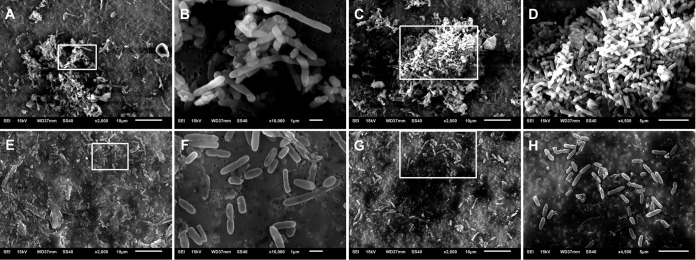
Scanning electron micrographs of *P. aeruginosa* CF_PA39 grown under low fluid shear conditions (A to D) or high fluid shear conditions (E to H). Panels B and D represent magnifications of the areas indicated by the white boxes in panels A and C, respectively. Panels F and H represent magnifications of the areas indicated by the white boxes in panels E and G, respectively. The magnification and scale bars are shown below each picture. Images are representative of different biological repeats.

Scanning electron microscopy showed that for each of the biological replicates, LS conditions were characterized by the presence of numerous clusters of closely associated cells (Fig. 2A to D) next to planktonic cells. The sizes of these clusters ranged from small (containing dozens of cells; Fig. 2A and B) to extremely large (thousands of cells; Fig. 2C). Inside these clusters, frequent cell-to-cell contacts were observed (Fig. 2B and D). In contrast to these findings, no clusters of *P. aeruginosa* cells could be observed for samples under HS conditions (Fig. 2E to H). Consequently, all cells adopted a unicellular planktonic lifestyle under HS conditions (Fig. 2F and H). Remarkably, elongated *P. aeruginosa* cells were regularly observed under both LS (see Fig. S3A in the supplemental material) and HS (see Fig. S3B) conditions. However, because of the many cell clusters found under LS conditions, it was not possible to quantify this phenotype. In summary, these observations indicate that biofilm formation by *P. aeruginosa* CF_PA39 was dependent on the prevailing shear stress. Similar nonattached aggregates were found when *P. aeruginosa* PAO1 was grown in static cultures for 48 h (42). Furthermore, it was shown that these nonattached aggregates are highly resistant to antibiotics as well as to phagocytes, a condition which could be reversed by disrupting them mechanically (42). In this report, we describe an alternative to physical disruption, namely, the use of shear stress, potentially leading to enhanced antibiotic susceptibility as well as removal by phagocytes.

### Effect of shear stress on the transcriptome of *P. aeruginosa* CF_PA39 grown in artificial sputum medium.

In order to detect genes that were differentially expressed in response to shear stress during growth of *P. aeruginosa* CF_PA39 in ASM in the RWV bioreactor, an RNA sequencing approach was followed. A complete list of the transcriptomic data is provided in Table S1 in the supplemental material. Differential shear stress conditions induced subtle differences in *P. aeruginosa* CF_PA39 gene expression, as expression of the most highly upregulated gene (*trpB*) under LS conditions was only 2.89-fold greater than that seen under the HS conditions, while expression of the most downregulated gene (*PA1923*) was only 2.57-fold lower (see Table S1). Because of these subtle differences, all genes that were differentially regulated ≥1.50-fold and for which the differences were statistically significant (*P* < 0.05; false-discovery rate [FDR] < 0.05) were included. In total, 133 and 107 genes were up- and downregulated under the LS conditions compared to the HS conditions, respectively. In order to look at the global response to shear stress, differentially expressed genes were first grouped according to their clusters of gene ontology (COG) class and the most affected functional classes were identified. Genes involved in nucleotide transport and metabolism were overrepresented among both up- and downregulated genes under LS conditions (see Table S2). Besides this functional class, mainly genes involved in transcription and (especially) translation were upregulated under LS conditions, while those involved in carbohydrate transport and metabolism were downregulated (see Table S2). Although this approach allows the identification of greatly affected functional classes, the level of resolution is limited. Therefore, all differentially expressed genes were subjected to a BLAST search using the Pseudomonas Genome Database and classified into specific functional classes (PseudoCAP, COG, KEGG) based on the available gene information (see Table S1). Based on these data, a comparison of the more specific functional classes that were upregulated or downregulated under LS conditions could be made (Table 1). The majority of the genes upregulated under LS conditions (without considering the hypothetical function class) belonged to translation (13.53%) and transcriptional regulation (9.77%) as predicted in the previous analysis (Table 1). In addition to these two large functional classes, genes involved in stress response (4.51%), denitrification (3.76%), glycerol metabolism (3.76%), alginate biosynthesis (3.01%), glycine betaine biosynthesis (1.50%), cell division (1.50%), tryptophan biosynthesis (1.50%), and type II secretion (1.50%) were identified among the genes upregulated ≥1.50-fold under LS conditions and were not present in the list of genes downregulated ≥1.50-fold. In contrast, genes involved in phenazine biosynthesis (2.80%), type VI secretion system (2.80%), cell motility (2.80%), lipid transport and metabolism (2.80%), Psl exopolysaccharide biosynthesis (1.87%), and secreted factors (1.87%) were exclusively found among the downregulated genes (Table 1).

**TABLE 1:** Comparison of the proportions of functional classes that were represented among genes upregulated or downregulated ≥1.50-fold under low fluid shear versus high fluid shear conditions

Functional class	Upregulated		Downregulated	
	No. of genes^a^	%	No. of genes^b^	%
Alginate biosynthesis	4	3.01	0	0.00
Amino acid transport and metabolism	4	3.01	14	13.08
Antibiotic resistance and susceptibility	3	2.26	2	1.87
Aromatic compound catabolism	1	0.75	0	0.00
Carbohydrate transport and metabolism	6	4.51	5	4.67
Carbon compound catabolism	0	0.00	1	0.93
Cell cycle control, cell division, chromosome partitioning	2	1.50	0	0.00
Cell motility	0	0.00	3	2.80
Cell wall/membrane/envelope biogenesis	5	3.76	2	1.87
Coenzyme transport and metabolism	5	3.76	2	1.87
Denitrification (anaerobic respiration)	5	3.76	0	0.00
Energy production and conversion	12	9.02	12	11.21
Glycerol metabolism	5	3.76	0	0.00
Glycine betaine biosynthetic process from choline	2	1.50	0	0.00
Glycine betaine catabolism	0	0.00	1	0.93
Glyoxylate and dicarboxylate metabolism	1	0.75	0	0.00
Inorganic ion transport and metabolism	4	3.01	1	0.93
Intracellular trafficking, secretion, and vesicular transport	3	2.26	2	1.87
Iron metabolism	2	1.50	0	0.00
Iron uptake	1	0.75	0	0.00
Lipid A biosynthetic process	1	0.75	0	0.00
Lipid transport and metabolism	0	0.00	3	2.80
Nucleotide transport and metabolism	6	4.51	3	2.80
Phenazine biosynthesis	0	0.00	3	2.80
Phosphonate metabolism	0	0.00	1	0.93
Posttranslational modification, protein turnover, chaperones	5	3.76	2	1.87
Psl biosynthesis	0	0.00	2	1.87
Replication, recombination, and repair	2	1.50	2	1.87
Rhamnolipid biosynthesis	0	0.00	1	0.93
Secreted factors (toxins, enzymes, etc.)	0	0.00	2	1.87
Signal transduction mechanisms	1	0.75	2	1.87
Stress response	6	4.51	0	0.00
TonB-dependent receptors	0	0.00	1	0.93
Transcriptional regulation	13	9.77	5	4.67
Translation, ribosomal structure, and biogenesis	18	13.53	4	3.74
Transport of small molecules	1	0.75	2	1.87
Tryptophan biosynthesis	2	1.50	0	0.00
Type II secretion system	2	1.50	0	0.00
Type IV pilus biogenesis	0	0.00	1	0.93
Type VI secretion system	0	0.00	3	2.80
Unknown	36	27.07	39	36.45

a Data represent a total of 133 genes.

b Data represent a total of 107 genes.

**FIG 3:**
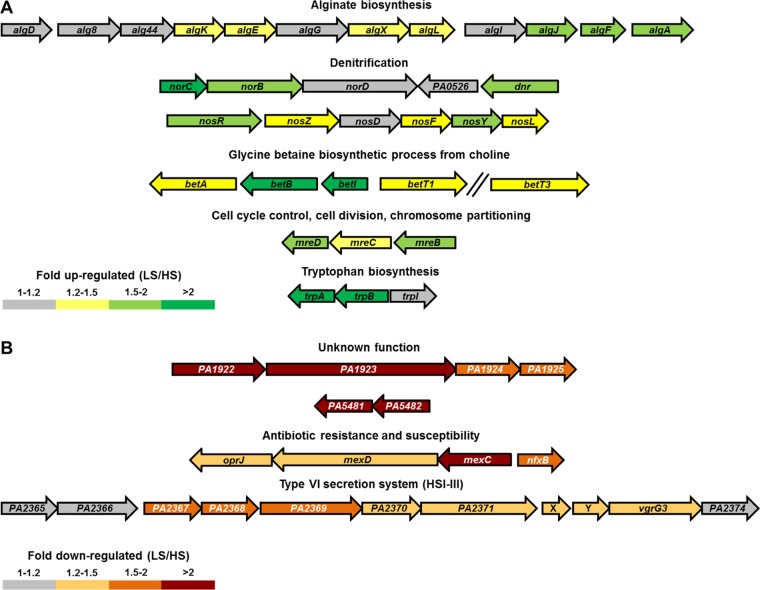
Overview of the genetic regions that contain upregulated (A) or downregulated (B) genes under low fluid shear versus high fluid shear conditions for the key affected functional classes. X and Y represent PA14 genes *PA14_33980* and *PA14_33970*, respectively, which are not present in the PAO1 genome. LS, low fluid shear. HS, high fluid shear. The // symbol indicates that this gene is located at a distant position in the genome. All adjacent genes that are transcribed in the same direction are considered to constitute an operon here.

The expression of a selection of genes (most differentially regulated and/or part of greatly affected functional classes) was confirmed via quantitative reverse transcription-PCR (qRT-PCR) (Table 2 and 3). Overall, gene expression results obtained with RNA sequencing and qRT-PCR overlapped for the selected genes (Table 2 and 3). Besides the genes belonging to the aforementioned functional classes, the differential expression levels of the *lasI* gene, encoding the 3-oxo-C_12_-HSL synthase, and *lasB*, encoding elastase, were confirmed, as both genes were slightly (<1.5-fold, *P* < 0.05) downregulated under LS conditions (Table 3). Interestingly, numerous genes that were upregulated under LS conditions were previously identified as being upregulated in CF sputum and/or chronic infection compared to planktonic cultures of the same *P. aeruginosa* strains (see Table S1 in the supplemental material) (43, 44). Furthermore, a large number of genes that were differentially regulated belonged to the same operon (Table 2 and 3; see also Table S1). By taking a lower cutoff value (≥1.20-fold differential expression; *P* < 0.05, FDR < 0.05), a number of operons containing genes of greatly affected functional classes can be distinguished (Fig. 3).

**TABLE 2:** Selection of genes that were upregulated under low fluid shear conditions compared to high fluid shear conditions according to RNA-Seq analysis and whose expression was confirmed via qRT-PCR analysis^a^

Gene	Product	Function	Operon	FC	
				RNA-Seq	qRT-PCR
*PA0036* (*trpB*)	Tryptophan synthase beta chain (EC 4.2.1.20)	Tryptophan biosynthesis; amino acid transport and metabolism	*PA0036* (*trpB*)–*PA0035* (*trpA*)	2.89	4.50 ± 1.58
*PA5374* (*betI*)	HTH-type transcriptional regulator BetI	Transcriptional regulation; glycine betaine biosynthetic process from choline; stress response	*PA5374* (*betI*)–*PA5372* (*betA*)	2.85	3.52 ± 0.60
*PA3584* (*glpD*)	Glycerol-3-phosphate dehydrogenase	Glycerol metabolism; energy production and conversion		2.29	2.92 ± 0.31
*PA0523* (*norC*)	Nitric oxide reductase subunit C (EC 1.7.99.7)	Denitrification (anaerobic respiration)	*PA0523* (*norC*)–*PA0525*	2.21	1.85 ± 0.13
*PA0579* (*rpsU*)	SSU ribosomal protein S21p	Translation, ribosomal structure, and biogenesis	*PA0579* (*rpsU*)–*PA0578*	2.17	2.95 ± 0.73
*PA3391* (*nosR*)	Nitrous oxide reductase maturation protein NosR	Denitrification (anaerobic respiration)	*PA3391* (*nosR*)–*PA3396* (*nosL*)	1.94	2.84 ± 0.82
*PA3550* (*algF*)	Alginate o-acetyltransferase AlgF	Alginate biosynthesis	*PA3540* (*algD*)–*PA3551* (*algA*)	1.83	1.94 ± 0.29
*PA4481* (*mreB*)	Rod-shape-determining protein MreB	Cell cycle control, cell division, chromosome partitioning	*PA4481* (*mreB*)–*PA4479* (*mreD*)	1.80	2.26 ± 0.30
*PA3551* (*algA*)	Mannose-1-phosphate guanylyltransferase (GDP) (EC 2.7.7.22)/mannose-6-phosphate isomerase (EC 5.3.1.8)	Alginate biosynthesis	*PA3540* (*algD*)–*PA3551* (*algA*)	1.65	2.01 ± 0.13

a FC, fold change; EC, enzyme class; HTH, helix-turn-helix; SSU, small subunit.

**TABLE 3:** Selection of genes that were downregulated under low fluid shear conditions compared to high fluid shear conditions according to RNA-Seq analysis and whose expression was confirmed via qRT-PCR analysis^a^

Gene	Product	Function	Operon	FC	
				RNA-Seq	qRT-PCR
*PA0121*	Transcriptional regulator, GntR family	Transcriptional regulation		−2.39	−2.19 ± 0.50
*PA5481*	Inhibitor of vertebrate lysozyme precursor	Periplasmic protein	*PA5482–PA5481*	−2.20	−2.26 ± 1.02
*PA3372*	Metal-dependent hydrolases of β-lactamase superfamily I; PhnP protein	Phosphonate metabolism	*PA3384* (*phnC*)–*PA3372*	−2.10	−5.54 ± 2.95
*PA1922*	Colicin I receptor precursor	TonB-dependent receptors; inorganic ion transport and metabolism	*PA1922–PA1925*	−2.07	−2.37 ± 0.46
*PA4599* (*mexC*)	Multidrug efflux RND membrane fusion protein MexC	Antibiotic resistance and susceptibility	*PA4599* (*mexC*)–*PA4597* (*oprJ*)	−2.04	−2.39 ± 0.94
*PA4171*	ThiJ/PfpI family protein	Unknown	*PA4171–PA4172*	−1.99	−3.40 ± 0.52
*PA4600* (*nfxB*)	Transcriptional regulator NfxB	Transcriptional regulation; antibiotic resistance and susceptibility		−1.65	−1.65 ± 0.64
PA5040 (*pilQ*)	Type IV pilus biogenesis protein PilQ	Type IV pilus biogenesis; cell motility	*PA5044* (*pilM*)–*PA5040* (*pilQ*)	−1.59	−1.28 ± 0.32
*PA4190* (*pqsL*)	2-Octaprenyl-3-methyl-6-methoxy-1,4-benzoquinol hydroxylase (EC 1.14.13)	Energy production and conversion		−1.50	−1.14 ± 0.48
*PA4209* (*phzM*)	Phenazine-specific methyltransferase PhzM	Phenazine biosynthesis		−1.45	−2.14 ± 0.77
*PA3724* (*lasB*)	Vibriolysin, extracellular zinc protease (EC 3.4.24.25); pseudolysin, extracellular zinc protease (EC 3.4.24.26)	Secreted factors (toxins, enzymes, alginate)		−1.31	−1.21 ± 0.16
*PA1432* (*lasI*)	*N*-Acyl-l-homoserine lactone synthetase LasI	Quorum sensing		−1.28	−1.07 ± 0.27

a FC, fold change; RND, resistance-nodulation-cell division superfamily; EC, enzyme class.

### Genes upregulated under low fluid shear conditions.

**FIG 4:**
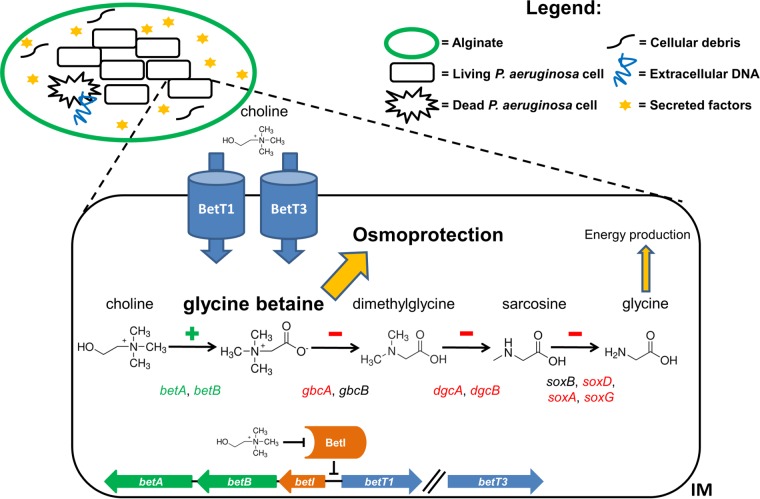
Hypothetical model of the adaptation of *P. aeruginosa* CF_PA39 to the low fluid shear conditions at the level of osmoprotection. Under the low fluid shear conditions, *P. aeruginosa* forms biofilms of closely associated cells that are surrounded by alginate layers. The production of several secreted molecules, as well as extracellular DNA and cell debris from dying cells, creates a local hyperosmotic environment. In order to protect itself against this hyperosmotic condition, *P. aeruginosa* imports choline via the BetT1 and BetT3 transporters, thereby releasing repression of the *betIBA* operon (and of choline transporter genes *betT1* and *betT3*) by the BetI repressor, and switches on the genes that are required for glycine betaine biosynthesis, using choline as a substrate. At the same time, the majority of genes involved in the catabolism of glycine betaine to glycine are downregulated, leading to an accumulation of the osmoprotectant glycine betaine. Genes highlighted in green were found to be upregulated whereas genes highlighted in red were found to be downregulated under low fluid shear versus high fluid shear conditions. The genes involved in the biosynthetic process proceeding from choline to glycine betaine are shown at the bottom. Glycine betaine biosynthetic genes *betA* and *betB* are shown in green, the *betI* repressor gene is shown in orange, and the choline transporter genes are shown in blue. The BetI protein is represented by an orange open cylindrical shape. The // symbol indicates that this gene is located at a distant position in the genome. IM, inner membrane.

Under LS conditions, the *algD* (alginate biosynthesis), *norCBD*, *nosRZDFYL* (denitrification), *betIBA* (glycine betaine biosynthesis from choline), *mreBCD* (cell cycle control, cell division, and chromosome partitioning), and *trpBA* (tryptophan biosynthesis) operons contained at least two genes upregulated ≥1.50-fold (Fig. 3A). Accordingly, the *P. aeruginosa* CF_PA39 strain formed robust biofilms under LS conditions, in agreement with our previous study (*P. aeruginosa* PAO1 grown in LB under LS conditions) (22). In contrast, under high fluid shear conditions, no clusters of cells could be observed, as all cells appeared to be unicellular and planktonic (Fig. 2). These phenotypic results matched with the transcriptomic data, since the exopolysaccharide alginate (encoded by the *algD* operon) is involved in *P. aeruginosa* biofilm formation (45–47). In addition to alginate biosynthesis, a prominent role for genes involved in denitrification was observed under LS conditions. More specifically, *norB*, *norC* (part of the *norCBD* operon), *nosR*, and *nosY* (part of the *nosRZDFYL* operon) were identified. This indicates that accumulation of nitric oxide (NO) is avoided during growth under LS conditions, since the *nor* (NO reductase) and *nos* (nitrous oxide reductase) genes are involved in the reduction of NO to N_2_*.* Furthermore, the gene encoding the transcriptional regulator Dnr (dissimilative nitrate respiration regulator) was found to be upregulated under LS conditions. Dnr is activated in the presence of NO, and the expression of *nirSMC* (part of the *nirSMCFDLGHJEN* operon), *nirQ*, *norCB*, and *nosZ* was found to be dependent on this regulator during growth under anaerobic conditions (48). In addition, the *anr* (fumarate and nitrate reduction regulatory protein) gene, which orchestrates the *P. aeruginosa* response to anaerobic conditions, was slightly (1.24-fold change), but significantly (*P* < 0.05, FDR < 0.05), upregulated during growth under LS conditions. These data indicate that *P. aeruginosa* CF_PA39 experiences microaerobic conditions during growth under LS conditions in ASM. In fact, our previous study showed that growth of *P. aeruginosa* PAO1 in LB led to a decreased oxygen transfer rate under LS conditions compared to that seen with a control under higher fluid shear conditions (23). Such microaerobic or anaerobic conditions could occur because of the low mixing capacities of the viscous ASM under LS conditions and/or locally inside the alginate-enclosed biofilms. A recent study strongly indicated that *P. aeruginosa* respires anaerobically in CF sputum via denitrification since an initial increase in the nitrous oxide (N_2_O) level was followed by a decrease after 6 h of monitoring in freshly expectorated CF sputum (49). Moreover, the addition of nitrate to LB medium, yielding physiological nitrate levels, resulted in increased growth rates of *P. aeruginosa* PAO1 as well as clinical *P. aeruginosa* CF isolates under anoxic conditions that were comparable to those observed in CF lungs and sputum (50). Genes involved in denitrification were not found to be differentially regulated during growth of *P. aeruginosa* PAO1 in LB medium under LS conditions (23), which was most probably due to the low nitrate levels in this medium. However, a recent study has shown that nitrate levels in ASM (identical to the ASM used here, except for the addition of 3 µg/ml ferritin) are comparable to those found in CF sputum samples (in the low millimolar range) (51). Another important pathway that was induced under LS conditions was the biosynthesis of glycine betaine. This compound can be synthesized from choline, via the intermediate betaine aldehyde, by means of the enzymes BetA (choline dehydrogenase) and BetB (betaine aldehyde dehydrogenase). Under LS conditions, the *betI* (encoding a transcriptional repressor whose repression activity is abolished in the presence of choline) and *betB* genes were among the most highly upregulated genes. In contrast to this finding, six of eight genes involved in catabolism of glycine betaine were downregulated ≥1.20-fold (*P* < 0.05, FDR < 0.05) under LS conditions. Interestingly, the *betI* and *betB* genes were found to be among the highest upregulated genes in one study in which the gene expression of *P. aeruginosa* in CF sputum *in vivo* was compared to that of a planktonic grown pool of isogenic isolates (44) and in another study in which *P. aeruginosa* gene expression shared under three chronic conditions (tumor, burn wound, and CF) was compared to that measured in the planktonic stage of growth (43). From our gene expression results, it can be deducted that the choline degradation pathway is pushed toward glycine betaine accumulation and not toward energy production via further catabolism of this compound, which is another option for *P. aeruginosa* as discussed by Wargo (52). Glycine betaine is an important osmoprotectant for many bacteria and is produced in response to stress conditions. We have developed a hypothetical model for the role of this important molecule under LS and CF conditions (Fig. 4). In robust biofilms, in which *P. aeruginosa* cells are enclosed by dense layers of alginate, a hyperosmotic situation could occur because of the local accumulation of cellular debris, extracellular DNA, and secreted factors such as secondary metabolites and enzymes. It has been previously shown that such higher-osmolarity conditions are present in *E. coli* biofilms compared to planktonic cultures (53). A higher-osmolarity situation, especially in the densest zones of biofilms, could occur, as it has been shown that diffusion of solutes in these zones is significantly slower than that seen in less-dense zones of *Stenotrophomonas maltophilia* biofilms (54, 55). Furthermore, the diffusion coefficients for several solutes in biofilms have been determined and appear to be significantly reduced compared to those in aqueous solutions. In general, it has been found that the effective diffusion coefficient for light gases (oxygen, NO, carbon dioxide) is about 40% reduced in biofilms compared to water, whereas the diffusion coefficient for most organic solutes in biofilms is reduced by 75% compared to aqueous solutions, most likely due to the presence of extracellular polymers, high bacterial cell density, and cellular debris (56). In order to respond to high-osmolarity conditions, *P. aeruginosa* is able to import choline from the environment and to catabolize it to obtain glycine betaine. Indeed, the *betT1* and *betT3* genes, which encode high-affinity choline transporters, were significantly (≥1.30-fold; *P* < 0.05, FDR < 0.05) upregulated under LS conditions, in addition to the glycine betaine biosynthetic genes and in contrast to the glycine betaine catabolic genes (see Table S1 in the supplemental material). An important reason why genes involved in glycine betaine biosynthesis were not upregulated under LS conditions in the previous RWV study (23) is most probably the absence of a choline source in the growth medium. The ASM used in the current study contained egg yolk, which is a major source of (phosphatidyl) choline. In their study, Son et al. (44) stated that (lung) phosphatidylcholine induced the expression of genes involved in fatty acid degradation, choline degradation, and glycerol metabolism. Furthermore, it was shown that expression of several lipases and phospholipases was induced in the presence of phosphatidylcholine. These phospholipases could cleave phosphatidylcholine, generating fatty acids, choline, and glycerol. Although increased expression of the phospholipase genes was not observed under LS conditions, genes involved in glycerol uptake (*glpF*) and metabolism (*glpK*, *glpD*) were upregulated ≥1.50-fold under LS conditions. Recently, it was shown that CF-adapted *P. aeruginosa* isolates utilize glycerol as a carbon source more efficiently than nonadapted isolates (57). In addition, mutation of the *glpD* gene (encoding a glycerol-3-phosphate dehydrogenase) resulted in lower levels of alginate production, indicating that the glycerol catabolic pathway is indispensable for full virulence of *P. aeruginosa* in chronic CF infection (57).

Besides the roles of glycine betaine biosynthesis and glycerol metabolism, cell division appears to play an important role under LS conditions since the *mreBCD* genes were upregulated under those conditions. These genes are involved in maintenance of the characteristic rod-like cell shape of bacteria through cell division (58). Recently, it was also observed that *mreB* (the most highly upregulated gene in the operon) is involved in osmotolerance in *Escherichia coli* (59). Furthermore, mutation of this gene appears to result in impairment of the correct localization of type IV pili and hence greatly affects the motility of *P. aeruginosa* (60). In the present study, we regularly observed elongated cells, most probably as a consequence of a defect in cell division since the characteristic shape of the septum was still visible on the surface of these cells (see Fig. S3 in the supplemental material). Such elongated cells were observed in another study when *P. aeruginosa* PAO1 was grown under anaerobic conditions (61). Furthermore, it was shown in the same study that upon mutation of *nirS*, required for the reduction of nitrite to NO, or addition of a NO antagonist to the culture medium, this phenotype was no longer observed and less-robust biofilms were formed. These data indicate that *P. aeruginosa* responds to anaerobic conditions by changing its cell shape. In addition, two studies described the elongation/filamentation of *Pseudomonas putida* cells when this bacterium was grown at low but not at high shaking speed (62, 63). Proteomic analysis of *P. putida* cultures grown at low shaking speed indicated that the elongated cell shape was most probably adopted as a survival strategy under the oxygen-limited conditions that are inherent in a lower shaking speed (62). Although we observed elongated cells under both LS and HS conditions, it was impossible to quantify this phenotype, since many bacterial cells were grouped together in tight clusters under LS conditions. Finally, the two genes of the *trpBA* operon, necessary for tryptophan biosynthesis, were among the four most highly upregulated genes under LS conditions (≥2.0-fold). In agreement with this observation, two other RWV studies have identified a role for tryptophan metabolism under LS conditions. More specifically, the *trpD* gene was found among the 68 upregulated genes when *S. enterica* serovar Typhimurium was grown under LS conditions compared to control conditions (higher fluid shear) (64). In a second study, a tryptophan permease-encoding gene was indispensable for the increased adherence of adherent-invasive *E. coli* to cell cultures under LS conditions (65). A more recent study showed that upregulation of the *trpD* gene under LS conditions compared to the control conditions is not ubiquitous among all genera of the *Enterobacteriaceae* family (66). The *in vivo* importance of the *trpBA* operon is less obvious, since these genes were upregulated in CF sputum in one study (44) but were downregulated in two others in either CF sputum (67) or CF-sputum-containing medium (68) compared to laboratory media.

### Genes downregulated under low fluid shear conditions.

Among the genes downregulated under LS conditions, many genes of unknown function were found, in addition to genes involved in cell motility, Psl biosynthesis, phenazine biosynthesis, the type VI secretion system, and multidrug resistance. All genes of the *PA1922-PA1925*, *PA3370-PA3371*, and *PA5482-PA5481* operons were found to be downregulated ≥1.50-fold, although their function(s) is unknown (Fig. 3B). In addition, the *mexC-oprJ* and *PA2365-PA2374* operons encoding the MexCD-OprJ multidrug efflux pump and the Hcp secretion island III (HSI-III) type VI secretion system, respectively, contain several genes that were downregulated ≥1.20-fold (Fig. 3B). *PA1922*, a TonB-dependent receptor gene that is part of the *P. aeruginosa* core genome (69), shares homology with the colicin I receptor of *Escherichia coli* (70). Previously, we have shown that deletion of the TonB-dependent receptor genes occurs frequently during adaptation of *P. aeruginosa* to the CF lung environment (13). Two divergent hypotheses can explain this observation. First, the biofilm lifestyle of *P. aeruginosa* in the CF lung could reduce selection pressure, leading to the loss of these genes. Second, pyocins, known to enter *P. aeruginosa* cells via TonB-dependent receptors (71–75), could select for their deletion. Therefore, TonB-dependent genes may be downregulated under biofilm-like rather than planktonic conditions due to rewiring of the regulatory networks that control expression of these genes. In agreement with this, no TonB-dependent receptor-encoding genes were detected among the upregulated genes under LS conditions. Although the function of the genes in the *PA5482-PA5481* operon remains to be elucidated, they have been associated with acute infection, since their expression was elevated in a non-CF pneumonia isolate of the Liverpool epidemic strain (LES) compared to a chronic CF LES isolate during growth in LB medium (76). With regard to genes of known function, it appeared that those involved in motility were downregulated under LS conditions. These genes are associated with the planktonic lifestyle of *P. aeruginosa* and have been shown to be prone to deletion during adaptation to the CF lung environment (77). More specifically, the *pilQ* gene, necessary for the formation of type IV pili, was significantly downregulated under LS conditions. Mutations in this gene have been frequently observed during *P. aeruginosa* colonization of the CF lung (9, 78). Several genes involved in phenazine biosynthesis were downregulated under LS conditions. This finding is in contrast with a previous study (79) where it was shown that the concentration of phenazines is positively correlated with the presence of ferrous iron in CF sputum. Ferrous iron concentrations were found to be higher in sputum from patients with deteriorating lung functions, most probably because of the microaerobic or anaerobic conditions encountered by *P. aeruginosa* in this environment. The results from the latter study suggest that phenazine biosynthesis is associated with the biofilm rather than with the planktonic lifestyle of *P. aeruginosa*. However, another study (80) showed that the *phzI* gene cluster is more highly expressed during planktonic growth whereas *phzII* almost exclusively contributed to phenazine biosynthesis in colony biofilms. Since only a draft genome of *P. aeruginosa* CF_PA39 is available, it was not possible to determine if both phenazine gene clusters were downregulated under LS conditions. Nevertheless, the *phzM* and *phzS* genes, which are exclusive to the *phzI* gene cluster, were downregulated under LS conditions 1.45-fold and 1.30-fold, respectively, indicating that *phzI* is expressed at a lower level under those conditions. Interestingly, two genes of the *psl* gene cluster that were found to be upregulated during growth of *P. aeruginosa* PAO1 under LS conditions (22) were downregulated ≥1.50-fold under LS conditions in the study conducted here. It is noteworthy that the relevance of this upregulation relative to Psl biosynthesis is low since *P. aeruginosa* CF_PA39 has a 3,376-bp deletion in the gene cluster comprising *pslAB* (13). However, it is interesting that the genes involved in Psl biosynthesis and in alginate production are differentially regulated. Recently, it was shown that the AmrZ transcription factor differentially regulates the two operons, as it represses the *psl* operon via binding to the *pslA* promoter region while activating the alginate biosynthetic operon (81). In *P. aeruginosa* CF_PA39, the *amrZ* gene appeared to be slightly but significantly upregulated in response to growth under low fluid shear conditions (see Table S1 in the supplemental material). However, the regulatory role of AmrZ in this strain needs to be investigated since the *psl* promoter region has a genetic architecture completely different from that seen with *P. aeruginosa* PAO1. Interestingly, several genes of the HSI-III type VI secretion system were significantly downregulated under LS conditions (Fig. 3). *P. aeruginosa* CF_PA39 has a PA14-like HSI-III type VI secretion system, including the *PA14_33980* and *PA14_33970* genes. In PA14, it has been shown that MfvR and LasR negatively regulate HSI-I while positively regulating HSI-II and HSI-III (82). In accordance to this, *mvfR* was slightly downregulated under LS conditions (see Table S1). Finally, the genes encoding the multidrug efflux pump MexCD-OprJ, as well as its transcriptional repressor gene, *nfxB*, were downregulated. Although it has been shown that this efflux system can be upregulated under envelope stress conditions in an *algU*-dependent way (83), no differential expression of the *algU* gene was observed in this study (see Table S1). It was shown in a recent paper that *nfxB* is essential for optimal fitness of *P. aeruginosa* PAO1 and PA14 during growth in MOPS (morpholinepropanesulfonic acid)-sputum medium (84). In addition, that report mentioned that several efflux genes were required for the fitness of *P. aeruginosa* grown in sputum, depending on the strain studied, hence indicating that the role of these genes in the fitness of a specific *P. aeruginosa* strain depends on the genetic framework of which they are part. The *nfxB* gene of *P. aeruginosa* CF_PA39 does not contain any frame shifts or preliminary stop codons. Furthermore, we did not detect any mutations that are known to perturb NfxB repressor activity (85) in screening the full-length *nfxB* gene (600 bp), indicating that the observed upregulation of MexCD-OprJ was not due to a defective *nfxB* gene.

### Comparison of the transcriptomic responses of *P. aeruginosa* PAO1 and *P. aeruginosa* CF_PA39 to LS conditions.

In comparisons of a previous study in which *P. aeruginosa* PAO1 was grown in LB medium at 28°C (23) to this study, only 18 genes were differentially regulated under LS conditions in the two studies (see Table S5 in the supplemental material). All of these genes, except *PA0835*, were upregulated under LS conditions. Interestingly, *glpD* was the most highly upregulated gene during growth of *P. aeruginosa* PAO1 under LS conditions, while the current study showed that it was among the six most highly upregulated genes during growth of *P. aeruginosa* CF_PA39 under LS conditions. In addition, *dnr*, involved in denitrification, was upregulated under LS conditions in both studies. The majority of commonly differentially regulated genes consisted of genes encoding ribosomal proteins that are typically under the control of *rpoD* (86). In fact, *rpoD* was slightly (1.39-fold) but significantly (*P* < 0.05, FDR < 0.05) upregulated under LS conditions in the current study, in agreement with the PAO1 study (23). Finally, heat shock genes *hslU* and *hslV*, involved in stress responses, were simultaneously upregulated in both studies. These results show that, although there was only a small overlap of differentially regulated genes in the two studies, several pathways that are related to biofilm formation (denitrification, glycerol metabolism, and stress response) were upregulated under LS conditions in both studies despite the use of different experimental conditions (28°C versus 37°C and LB versus ASM) and strains (*P. aeruginosa* PAO1 versus *P. aeruginosa* CF_PA39).

### Role of small RNAs in the shear stress response.

Small RNAs (sRNAs) have been shown to be important regulators of gene expression in many bacteria, since they are involved in posttranscriptional modification of mRNA transcripts (87, 88). They can regulate mRNA levels either positively, by enhancing ribosome binding, or negatively, by blocking the ribosome-binding site and/or enhancing RNase E-mediated degradation of the target transcript (89). The interaction between the sRNA and a target mRNA can be mediated through the Hfq RNA chaperone, and this has been described in *E. coli*, *S. enterica*, and *P. aeruginosa*, among others species (90, 91). Interestingly, *hfq*, as well as other genes belonging to the Hfq regulon, has been observed to be greatly involved in the response to LS conditions in different studies involving *S. enterica* serovar Typhimurium, *P. aeruginosa*, and *S. aureus* (22, 23, 92–94). In accordance with the growth of *P. aeruginosa* PAO1 under LS conditions in LB medium seen in our previous study (23), *hfq* was slightly (1.3-fold) upregulated (*P* < 0.05, FDR < 0.05) under LS conditions in the current study. The expression of experimentally validated sRNA genes (95) as well as of newly predicted small RNA genes based on secondary structure prediction and RNA sequencing analysis for *P. aeruginosa* CF_PA39 was determined under LS conditions and HS conditions. In total, three sRNA genes (*SPA0071*, *SPA0102*, and the P34 gene) were found to be statistically significantly (*P* < 0.05, FDR < 0.05) upregulated under LS conditions (Table 4) whereas four sRNA genes (the sRNA10, *SPA0117*, P8, and *SPA0003* genes) were downregulated (Table 5). All but one of these differentially regulated sRNA genes are present in the PAO1 genome, and their exact positions are indicated in Tables 4 and 5. Both the sRNA *SPA0102* gene and the adjacent *PA3162* gene are upregulated under LS conditions. In contrast, the sRNA P8 gene and the adjacent *PA1030* gene are both downregulated. Interestingly, the predicted *de novo* sRNA10 gene was highly (2.35-fold) downregulated under LS conditions, while the *PA3966* and *PA3967* genes, which are in close proximity to this sRNA gene, were upregulated ≥1.50-fold. One of the future objectives is to test whether these sRNAs regulate those adjacent genes. Interestingly, the sRNA P34 gene, which was more highly expressed under LS conditions, was also found to be upregulated in stationary-phase planktonic cultures and static biofilms of *P. aeruginosa* PA14 grown in LB (96).

**TABLE 4:** List of small RNA genes that were significantly upregulated^a^ under low fluid shear conditions compared to high fluid shear conditions

Small RNA gene	Length (bp)	Position in PAO1 genome^b^	Experimentally validated^c^	Fold change (RNA-Seq)
*SPA0071*	201	IR *PA0805–PA0806*	Yes	1.94
*SPA0102*	301	IR *PA3162* (*rpsA*)–*PA3163* (*cmk*); overlapping *cmk*	Yes	1.78
P34	399	IR *PA5181–PA5182*	Yes	1.50

a Upregulated ≥1.50-fold; *P* < 0.05, FDR < 0.05.

b IR, intergenic region.

c Listed by Tsai et al. (95).

**TABLE 5:** List of small RNA genes that were significantly downregulated^a^ under low fluid shear conditions compared to high fluid shear conditions

Small RNA gene	Length (bp)	Position in PAO1 genome^b^	Experimentally validated^c^	Fold change (RNA-Seq)
sRNA10^d^	202	IR *PA3964–PA3965*	No	−2.35
*SPA0117*	201	IR *PA3049* (*rmf*)–*PA3050* (*pyrD*); overlapping both genes	Yes	−1.94
P8	78	IR *PA1030–PA1031*	Yes	−1.85
*SPA0003*	137	IR *PA2729–PA2730*	Yes	−1.58

a Downregulated ≥1.50-fold; *P* < 0.05, FDR < 0.05.

b IR, intergenic region.

c Listed by Tsai et al. (95).

d This small RNA gene was *de novo* predicted in this study.

### Quorum sensing molecules are produced at slightly higher levels in response to shear stress.

**FIG 5:**
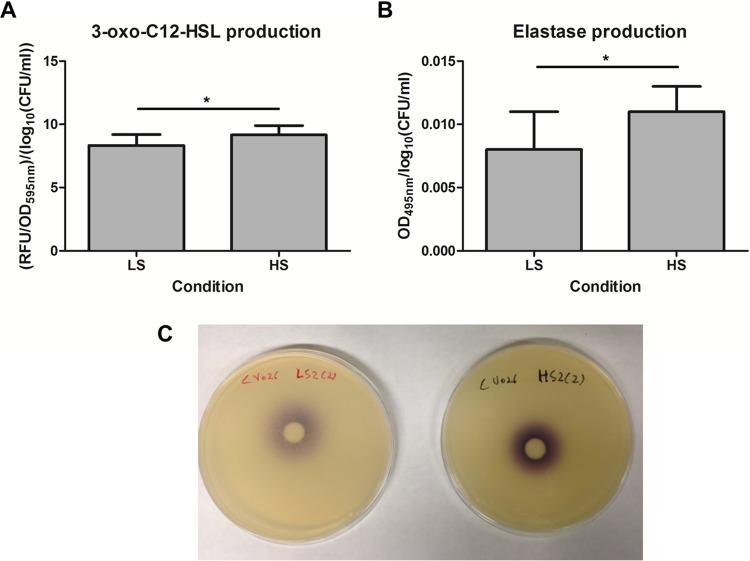
Production of QS molecules and elastase during growth under different shear stress conditions. (A) 3-Oxo-C_12_-HSL production. (B) Elastase production. (C) Production of short-chain (C_4_-C_8_) AHL molecules by *P. aeruginosa* CF_PA39 grown under low fluid shear (plate shown on the left) or high fluid shear (plate shown on the right) conditions. The picture shown here is representative of the results from all three technical replicates of each biological replicate. LS, low fluid shear. HS, high fluid shear. RFU, relative fluorescence units. *, *P* < 0.05.

In agreement with the RNA sequencing and qRT-PCR data, a subtle but statistically significant difference between LS conditions and HS conditions was found in the levels of 3-oxo-C_12_-HSL production (Fig. 5A). In addition, elastase production was significantly higher under the HS conditions than under the LS conditions (Fig. 5B). For the qualitative determination of levels of short-chain *N*-acylhomoserine lactones (AHLs) (e.g., C_4_-HSL), *Chromobacterium violaceum* indicator strain CV026, which produces the deep purple compound violacein in the presence of short-chain AHLs, was used. A deep purple color was observed under HS conditions, whereas only a light purple color was present on plates containing supernatant from LS replicates, indicating that the production of short-chain AHLs was higher under HS than under LS conditions (Fig. 5C). The elevated production of QS molecules and elastase under HS conditions is in contrast with the results of our previous RWV study (22) in which *P. aeruginosa* PAO1 was grown in LB medium. Nevertheless, the different genetic background, the complexity of the medium utilized in this study, and, most importantly, the highly restructured regulatory network as a consequence of chronic adaptation to the CF lung condition are suggested to have caused this discrepancy. Interestingly, the dispersal variants identified after 9 days of growth in the study of Woo et al. (33) produced significantly larger amounts of the 3-oxo-C_12_-HSL and short-chain AHLs as well as elastase. Similarly, the subtle increase in the production of QS molecules and the QS-dependent product elastase observed under HS conditions in this study might have been the result of a slightly enriched biofilm dispersal population that had adopted a more planktonic lifestyle. Taken together, these data indicate that a phenotypic variant might have emerged as a result of genotypic diversification, similarly to the situation in the CF lung.

### Conclusion.

In this study, the response of a transmissible CF-adapted *P. aeruginosa* isolate to differential shear stress conditions was studied at the transcriptomic as well as the phenotypic level in medium resembling CF sputum. Following an RNA sequencing approach, genes involved in alginate biosynthesis, denitrification, cell shape determination, glycine betaine biosynthesis, glycerol metabolism, and tryptophan biosynthesis were found to be upregulated under LS conditions, which presumably contributed to the observed biofilm formation. In contrast, genes involved in motility, phenazine biosynthesis, type VI secretion, and multidrug efflux, as well as many hypothetical genes, were downregulated. Overall, these transcriptomic results are in agreement with the SEM observations that revealed the formation of robust biofilms only under LS conditions. Furthermore, a number of sRNA genes might play a role in this switch from the biofilm to the planktonic life cycle. In comparisons of the shear stress response observed in the study conducted here to that observed in a previous study that used *P. aeruginosa* PAO1 in LB medium, both similarities and differences were observed. Commonalities between the two studies include the formation of self-aggregating biofilms under low shear conditions, as well as the induction of genes involved in alginate synthesis, stress response, and responses to low oxygen conditions. Interestingly, the use of a highly adapted *P. aeruginosa* CF isolate and growth medium directly relevant to the CF lung environment resulted in the induction of additional pathways that had previously been shown to play a role in the metabolism and virulence of this pathogen in the CF patient and that were not found in our previous study using a non-CF *P. aeruginosa* strain and LB medium. We hypothesize that the combination of physicochemical factors (such as fluid shear, viscosity, and nutritional content) and the relevant bacterial genetic background in the present study induced expression of phenotypic and molecular genetic traits in *P. aeruginosa* that have been observed previously *in vivo*. Finally, since high fluid shear conditions precluded the formation of CF-like biofilms by *P. aeruginosa*, the results presented in this study are promising with regard to future *in vivo* applications that would introduce shear stress with the aim of disrupting *P. aeruginosa* biofilms. To this end, the use of certain established types of physical therapy for CF patients, such as high-frequency chest wall oscillation or intrapulmonary percussive ventilation, could be considered. These techniques could possibly introduce shear stress in the lungs of CF patients, thereby causing *P. aeruginosa* to transition from a biofilm mode of growth to a planktonic lifestyle.

## MATERIALS AND METHODS

### Bacterial strains and culture conditions.

All bacterial strains used in this study are listed in Table S3 in the supplemental material. Bacterial overnight cultures were grown at 37°C in nutrient-rich lysogeny broth (LB) medium (Life Technologies), with continuous shaking at 200 rpm unless mentioned otherwise, prior to inoculation in ASM.

### Preparation of artificial sputum medium.

ASM was prepared as described by Fung et al. (68) without the addition of antibiotics. In order to obtain sterile ASM, stock solutions of bovine serum albumin (BSA), Casamino Acids (CAA), salts, diethylene triamine pentaacetic acid (DTPA), and salmon sperm DNA were sterilized by filtration prior to use. Furthermore, the porcine stomach mucin solution was autoclaved at 121°C for 15 min since the viscosity of this solution did not allow filter sterilization. Next, a 500-ml solution of ASM was prepared by combining all of the solutions mentioned above with a sterile egg yolk solution in a laminar flow cabinet. In contrast to the pH used in the study by Fung et al. (68), the pH of the ASM was adjusted to 6.8, in agreement with the study of Palmer et al. (67), in which this value was found to be representative of the slightly acidic conditions found in the CF lung. Sterility of the ASM was verified by plating 100 µl of this solution on LB, followed by incubation at 37°C for 72 h.

### Rotating wall vessel experiment.

An isolated mucoid colony of *P. aeruginosa* CF_PA39 was inoculated into 5 ml of LB medium and grown overnight with continuous shaking at 200 rpm. Next, the optical density at 600 nm (OD_600_) was adjusted to a value of 1.0 using LB medium and 1 ml of the adjusted bacterial culture was centrifuged at 8,000 × *g* to collect the cells. The resulting pellet was washed once with 1× phosphate-buffered saline (PBS) and resuspended in 1 ml of this buffer. Subsequently, this bacterial culture was diluted 1,000× in ASM and briefly (5 s) subjected to vortex mixing, and ~50 ml of this mixture was transferred to the RWV bioreactor (Synthecon Inc.) via the filling port with (high fluid shear [HS]) or without (low fluid shear [LS]) two glass beads (Merck Millipore) (diameter, 4 mm). Air bubbles were removed via the sampling ports using a 5-ml syringe. Finally, the RWV bioreactors were incubated at 37°C for 24 h at 25 rpm while the humidity of the incubator was maintained. The experiment was performed in biological triplicate.

### Determination of bacterial counts in the RWV.

In order to recover the entire bacterial population after 24 h of growth in the RWV bioreactor, about half of the bacterial culture volume was transferred to a 50-ml Falcon tube, while the other half was subjected to vortex mixing for 30 s inside the RWV bioreactor. In this way, the bacterial cells that were attached to the gas-permeable silicone membrane were included. Finally, the two volumes were pooled and briefly (5 s) subjected to vortex mixing. Serial dilutions of the biological triplicates corresponding to each condition and prepared in PBS were plated on LB medium, and colonies were quantified according to their phenotype after 24 h of growth at 37°C.

### Determination of bacterial counts in culture tubes.

*P. aeruginosa* CF_PA39 was grown in 5 ml of ASM or LB medium at 37°C at 220 rpm for up to 124 h, and the ratio of mucoid colonies to nonmucoid colonies was determined by plating serial dilutions on LB plates after 24 h of growth at 37°C.

### RNA isolation.

Multiple 2-ml aliquots of the bacterial cell culture, recovered from the RWV bioreactor as mentioned above, were centrifuged for 5 min at 8,000 × *g*. The resulting pellet was flash-frozen in liquid nitrogen and stored at −80°C until further processing. RNA was extracted using an SV total RNA isolation system (Promega). In summary, the thawed cell pellet was resuspended in 200 µl of a freshly prepared lysozyme solution (3 mg/ml in Tris-EDTA buffer) followed by 5 min of incubation at room temperature. Next, this mixture was divided into two smaller volumes. Upon cell lysis and addition of RNA dilution buffer, samples were centrifuged for 15 min at maximum speed and the clear supernatant was transferred to fresh 1.5-ml microcentrifuge tubes. This step was necessary to avoid blocking of the column since the lysates appeared to be viscous, most likely due to the presence of alginate and/or mucin. Upon the addition of ethanol and a washing step, an on-column DNase treatment was performed, followed by two washing steps. Finally, RNA was eluted in nuclease-free water. Prior to RNA sequencing and qRT-PCR, an additional Turbo DNase (Ambion) treatment was performed via two 30-min incubation steps in the presence of 1 µl of (2 U/µl) Turbo DNase. In a next step, the Turbo DNase-treated RNA was purified and concentrated using a RNA Clean and Concentrator kit (Zymo Research), allowing the recovery of total RNA (>17 nucleotides). RNA quantity was determined using a NanoDrop ND-1000 spectrophotometer (NanoDrop Technologies), while RNA quality was assessed via the use of an Agilent 2100 Bioanalyzer and an Agilent RNA 6000 Nano kit (Agilent Technologies). The removal of genomic DNA was verified via 35 cycles of PCR amplification (5 min at 95°C, followed by 35 cycles of 45 s at 95°C, 1 min at 55°C, and 1 min at 72°C and a final extension step of 7 min at 72°C) of the *uvrD* gene (forward primer, 5′ GTAGCGAGACCTACAACAAGGTTTC 3′; reverse primer, 5′ TGGACAGGCGCACTTCCT 3′) of *P. aeruginosa*.

### RNA sequencing and data analysis.

Ten micrograms of extracted total RNA was treated with a Ribo zero kit (Epicentre) to enrich for mRNA by removing the 16S and 23S rRNA. Paired-end libraries were prepared according to the *TruSeq RNA Sample Preparation Guide* (Illumina). The library preparation and Illumina RNA sequencing were performed by the use of BaseClear (Leiden, The Netherlands). Obtained reads were aligned using BWA software and the default parameters (97). Raw counts per gene were calculated based on the genome annotation of *P. aeruginosa* CF_PA39 (GenBank accession number NZ_JDVE00000000). Reads were allowed to map 50 bp upstream of the start codon or 50 bp downstream of the stop codon. Reads that were mapped to ribosomal or transporter RNA were removed from the raw count data to prevent bias in detecting differential expression. Differential expression was calculated using the edgeR package (version 3.2.4) (98) in BioConductor (release 3.0, R version 3.1.2), resulting in a fold change value and a corresponding *P* value corrected for multiple testing for each gene. Genes found to be differentially expressed (≥1.50-fold; *P* < 0.05, FDR < 0.05) were subjected to a BLAST search using the Pseudomonas Genome Database (99) in order to obtain information about their presence in other *P. aeruginosa* genomes, the functional classes that they belong to, their genetic organization, and related literature. A lower cutoff value (≥1.20-fold differential expression; *P* < 0.05, FDR < 0.05) was adopted only to complete the gene lists belonging to differentially regulated pathways.

For the detection of new sRNAs located in intergenic regions, the RNA-Seq reads were mapped via READemption pipeline 0.3.4 (using segemehl version 0.1.7 [100]) followed by coverage calculations. The sRNA prediction was conducted using ANNOgesic (S.-H. Yu, unpublished data). For this, positions with coverage at levels higher than 5 reads were combined to transcripts (gaps of a maximum of 5 nucleotides [nt] with lower coverage were accepted). Transcripts shorter than 20 nt or longer than 500 nt and transcripts that overlapped known genes in sense or anti-sense orientation were discarded. For the remaining candidates, the secondary structure was predicted by the use of RNAfold (part of the Vienna package [101]) and only sRNA candidates that were able to form a secondary structure were kept. The sRNAs were aligned against the sRNA entries in BSRD (Bacterial Small Regulatory RNA Database) (102) with BLAST 2/2/28+ (103), but none showed significant homology (i.e., an E value below 0.0001).

### Reverse transcription.

cDNA was prepared using an iScript cDNA synthesis kit (BioRad), starting from 1 µg of DNA-free total RNA. The resulting cDNA was diluted 5× prior to use in qRT-PCR. In order to verify efficient conversion of RNA to cDNA, a PCR was performed using primers (forward, 5′ ATGAACAACGTTCTGAAATTCTCTGCT 3′; reverse, 5′ CTTGCGGCTGGCTTTTTCCAG 3′) that allowed amplification of the *oprI* gene in a thermocycler (TC-412-Techne) under the following thermocycling conditions: 94°C for 5 min, followed by 35 cycles of 94°C for 45 s, 55°C for 45 s, and 1 min at 72°C and a final elongation step at 72°C for 10 min.

### Quantitative real-time PCR.

Since the aim of this study was to identify genes that are differentially regulated in response to shear stress and that could later serve as marker genes *in vivo*, we decided to design qRT-PCR primers that are able to anneal to target genes in various *P. aeruginosa* strains without any mismatches. All primers used in qRT-PCR amplification were designed via Primer3 (104) and are listed in Table S4 in the supplemental material. Amplification was performed in a 96-well plate, in which each well contained 25 µl of a volume consisting of 9.5 µl of nuclease-free water, 1 µl of each primer (10 µM), 12.5 µl of 2× iQTM SYBR Green supermix (BioRad), and, finally, 1 µl of template cDNA (5× diluted). The PCR amplification was performed using an iQ2 real-time PCR detection system (BioRad) and the following program: an initial cycle at 95°C for 3 min for denaturation and enzyme activation and then 40 cycles of 95°C for 10 s and 55°C for 60 s. Finally, melt curves were determined to identify primer dimer formation. qRT-PCR results were normalized against the *oprI* housekeeping gene encoding major outer membrane lipoprotein I. Fold changes were calculated using the method of Livak and Schmittgen (105). The experiment was performed in biological and technical triplicates.

### Scanning electron microscopy.

Bacterial cells were recovered from the RWV bioreactor as mentioned before and subsequently diluted 1,000× in PBS via serial dilutions. Next, 500 µl of each dilution was concentrated on a Nuclepore TrackEtch Polycarbonate membrane filter with 0.2 µm pore size, followed by two fixation steps of 20 min with a 3% (wt/vol) glutaraldehyde–0.15 M cacodylate solution. In a following step, the membrane was washed three times with the 0.15 M cacodylate wash solution and stored overnight at 4°C. Dehydration was obtained by rinsing the filter surface with an ascending series of ethanol concentrations (30%, 50%, 70%, 90%, 95%, and 100% [vol/vol] in Milli-Q water). The final 100% ethanol solution was replaced three times. Next, the ethanol solution was replaced with hexamethyldisilazane and this was repeated three times. Finally, the membrane filters were air-dried at room temperature in a desiccator overnight, taped onto a brass stub using carbon tape, and ultimately sputter coated with gold particles. SEM analysis was performed on a JEOL JSM-840 microscope (JEOL Ltd.) equipped with a secondary electron detector and a backscatter electron detector (point electronic GmbH) at a working distance of 37 mm and 15-kV acceleration.

### Quantification of 3-oxo-C_12_-HSL.

Supernatants of RWV cultures were obtained via centrifugation of 25 ml of culture medium at 10,000 × *g* for 10 min and stored as 2-ml aliquots at −20°C prior to use. An overnight culture of *E. coli* indicator strain MH155 (see Table S3 in the supplemental material), which produces green fluorescent protein (GFP) in the presence of 3-oxo-C_12_-HSL (106), was diluted to an OD_600_ of 1.0. Next, 100 µl of this diluted overnight culture and 200 µl of RWV culture supernatant were added to 5 ml of LB medium and incubated at 37°C for 24 h with continuous shaking at 200 rpm. Finally, 200 µl of the bacterial culture was transferred to a 96-well plate and the OD_595_ as well as relative fluorescence unit (RFU) values obtained at excitation and emission wavelengths of 485 nm and 527 nm, respectively, were determined using a Fluoroskan Ascent fluorometer (Thermo Scientific). Background-corrected RFU values were normalized to the OD_595_ of the reporter strain and to the log_10_ CFU value per milliliter for each replicate. The experiment was performed in biological and technical triplicates.

### Determination of elastase production.

The amount of extracellular elastase was assessed by means of a Congo red colorimetric assay (107). Defrost supernatant (100 μl) was added to glass test tubes containing 10 mg of elastin Congo red (Sigma-Aldrich) and 900 µl of 0.1 M Tris-HCl (pH 7.2). After 6 h of incubation at 37°C, the tubes were centrifuged (10 min, 10,000 × *g*) and 250 µl of supernatant was pipetted in a 96-well microtiter plate. Finally, the optical density at 495 nm was measured using a Multiskan Spectrum spectrophotometer (Thermo Scientific). Finally, background-corrected OD_495_ values were normalized to the log_10_ CFU value per milliliter for each replicate. The experiment was performed in biological and technical triplicates.

### Qualitative determination of short-chain AHLs.

LB plates (25 ml of LB agar) were covered with a 5-ml 0.6% LB top agar layer and 100 µl of *Chromobacterium violaceum* indicator strain CV026 (see Table S3 in the supplemental material), which produces violacein and is characterized by a deep purple color in the presence of short-chain *N*-acylhomoserine lactones (AHLs) (108). After this top agar layer was allowed to dry for 10 min in a laminar flow cabinet, a diffusion disk (Oxoid) containing 70 µl of culture supernatant was applied to the top of this soft agar by gently pressing the top of the disk by means of a forceps. Finally, the plates were incubated at 30°C for 24 h. The experiment was performed in biological and technical triplicates.

### Statistical analyses.

All experiments were performed in triplicate. A paired, two-tailed Student’s *t* test was applied to the data obtained from the bacterial count, 3-oxo-C_12_-HSL, and elastase experiments in order to detect differences between the LS and HS conditions. *P* values of <0.05 were considered to be statistically significant. Clusters of gene ontology (COG) classes enriched under either LS or HS conditions were identified by means of hypergeometric distribution.

## SUPPLEMENTAL MATERIAL

Figure S1  Relative abundances of the mucoid and nonmucoid morphotypes after growth of *P. aeruginosa* CF_PA39 in ASM in culture tubes over time. Download Figure S1, TIF file, 0.1 MB

Figure S2  Relative abundances of the mucoid and nonmucoid morphotypes after growth of *P. aeruginosa* CF_PA39 in LB medium in culture tubes over time. Download Figure S2, TIF file, 0.1 MB

Figure S3  Elongated *P. aeruginosa* cells observed under low fluid shear (A) and high fluid shear (B) conditions via scanning electron microscopy. Images are representative of different biological repeats. Elongated cells are indicated by white arrows. Red arrows indicate the putative septum that was formed during cell division. Download Figure S3, TIF file, 0.5 MB

Table S1  Overview of the RNA sequencing results for all coding sequences (CDS) and small RNA genes annotated in *P. aeruginosa* CF_PA39. Table S1, XLSX file, 1.5 MB

Table S2  Analysis of the relative proportions of COG-functional classes that were represented among differentially expressed genes. Table S2, XLSX file, 0.02 MB

Table S3  Bacterial strains used in this study. Table S3, DOCX file, 0.02 MB

Table S4  Primers used for qRT-PCR amplification. Table S4, DOCX file, 0.02 MB

Table S5  Genes that are differentially expressed in *P. aeruginosa* CF_PA39 and *P. aeruginosa* PAO1 during growth under LS versus HS conditions. Table S5, XLSX file, 0.01 MB
