# Supplementary figures and images for: Effect of Shear Stress on Pseudomonas aeruginosa Isolated from the Cystic Fibrosis Lung
Source: mBio. 2016 Aug 2;7(4):e00813-16. doi: 10.1128/mBio.00813-16 (PMC4981712; doi:10.1128/mBio.00813-16)

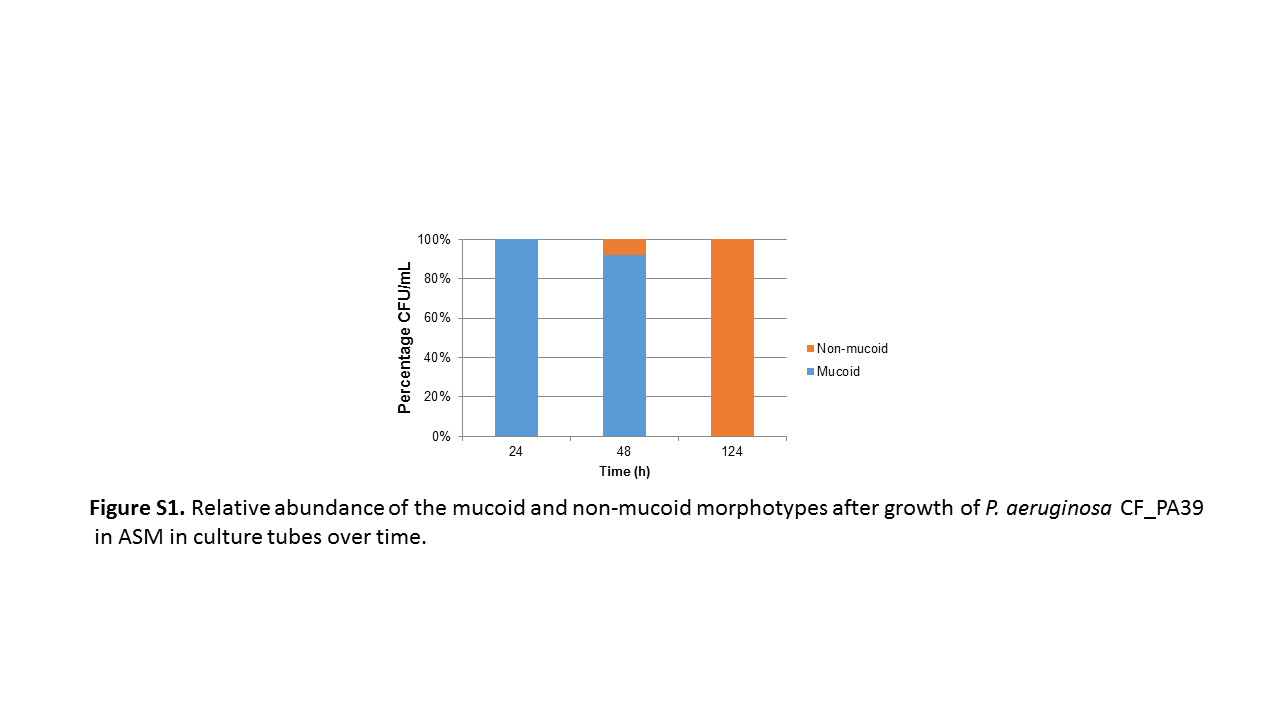

Supplement: Figure S1 — Relative abundances of the mucoid and nonmucoid morphotypes after growth of P. aeruginosa CF_PA39 in ASM in culture tubes over time. Download [file mbo004162926sf1.tif]

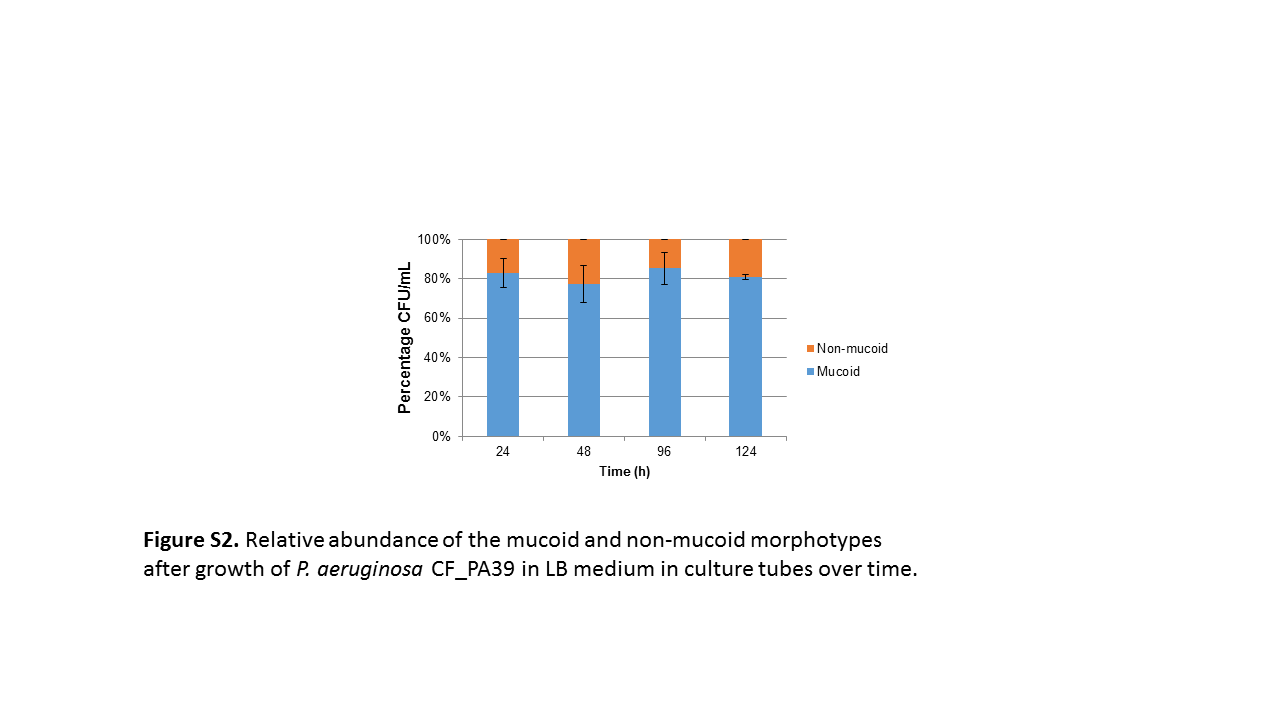

Supplement: Figure S2 — Relative abundances of the mucoid and nonmucoid morphotypes after growth of P. aeruginosa CF_PA39 in LB medium in culture tubes over time. Download [file mbo004162926sf2.tif]

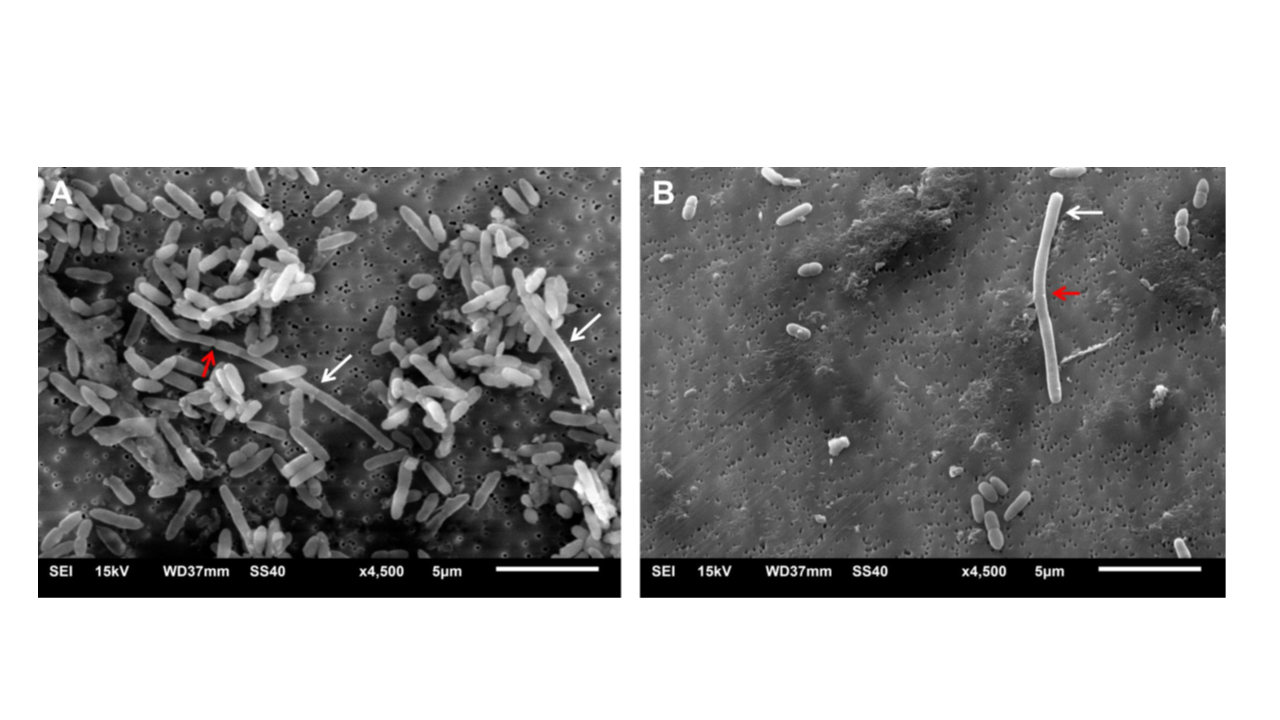

Supplement: Figure S3 — Elongated P. aeruginosa cells observed under low fluid shear (A) and high fluid shear (B) conditions via scanning electron microscopy. Images are representative of different biological repeats. Elongated cells are indicated by white arrows. Red arrows indicate the putative septum that was formed during cell division. Download [file mbo004162926sf3.tif]
