# Supplementary material for: Effect of Shear Stress on Pseudomonas aeruginosa Isolated from the Cystic Fibrosis Lung
Source: mBio. 2016 Aug 2;7(4):e00813-16. doi: 10.1128/mBio.00813-16 (PMC4981712; doi:10.1128/mBio.00813-16)
Supplement: Table S3 — Bacterial strains used in this study. [file mbo004162926st3.docx]

**Table S3.** Bacterial strains used in this study.

| Strain | Genotype | Reference |
| --- | --- | --- |
| CF_PA39 | *P. aeruginosa* CF isolate; MLST profile *acs*:118/*aro*:106/*gua*:85/*mut*:86/*nuo*:72/*pps*:73/*trp*:71 | Dingemans *et al*., 2014 |
| MH155 | *E. coli* strain carrying plasmid pMHLAS (pUCP22NotI-P*lasB*::*gfp*(ASV)P*lac*::*lasR*) | Hentzer *et al.*, 2004 |
| CV026 | *C. violaceum* mutant strain harboring a mini-Tn*5* insertion in the C6-HSL-encoding gene *cviI* (Sm^r^ mini-Tn5 Hg^r^ cviI::Tn5xylE Km^r^) | McClean *et al*., 1997 |
