# Supplementary material for: Effect of Shear Stress on Pseudomonas aeruginosa Isolated from the Cystic Fibrosis Lung
Source: mBio. 2016 Aug 2;7(4):e00813-16. doi: 10.1128/mBio.00813-16 (PMC4981712; doi:10.1128/mBio.00813-16)
Supplement: Table S4 — Primers used for qRT-PCR amplification. [file mbo004162926st4.docx]

**Table S4.** Primers used for qRT-PCR amplification.

| Primer | Sequence (5’→3’) | Amplicon size (bp) | Applicability to other genomes^a^ |
| --- | --- | --- | --- |
| trpB_F | CCTACTTCCAGCGCGACTAC | 118 | PAO1/LESB58/DK2/PA14/RP73/B136-33/PACS2 |
| trpB_R | CCGGTATGGTTCAGCTCCT |  |  |
| PA0121_F | CCACCAGCCATTTCGTCTAC | 88 | PAO1/LESB58/DK2/PA14/RP73/B136-33/PACS2/PA7 |
| PA0121_R | AGGGTTTCCTTGCTCAGCTT |  |  |
| norC_F | GCTTCAACACCTTCCTCCAG | 139 | PAO1/LESB58/DK2/PA14/RP73/B136-33/PACS2/PA7 |
| norC_R | TCGATCTTCGAGCTCCACTT |  |  |
| rpsU_F | GCCAGCCGTCAAAGTAAAAG | 122 | PAO1/LESB58/DK2/PA14/RP73/B136-33/PACS2/PA7 |
| rpsU_R | TGGGCTTCTCGTAGAACTCG |  |  |
| lasI_F | CGTGCTCAAGTGTTCAAGGA | 131 | PAO1/LESB58/DK2/PA14/RP73/B136-33/PACS2 |
| lasI_R | AAAACCTGGGCTTCAGGAGT |  |  |
| PA1922_F | CTGGAGCTGAGCCAGAAACT | 101 | PAO1/LESB58/DK2/PA14/RP73^*^/B136-33/PACS2 |
| PA1922_R | TAGGTGAAGTGCGTGTCGTC |  |  |
| oprI_F | AGCAGCCACTCCAAAGAAAC | 108 | PAO1/LESB58/DK2/PA14/RP73/B136-33/PACS2/PA7 |
| oprI_R | CAGAGCTTCGTCAGCCTTG |  |  |
| PA3372_F | TCCTCCAGACCCATTACCAC | 150 | PAO1/LESB58/DK2/PA14/RP73/B136-33/PACS2 |
| PA3372_R | GCTGGCTGAAATCGAGGAT |  |  |
| nosR_F | CTTCCGCGACCTCGACTAC | 136 | PAO1/LESB58/DK2/PA14/RP73/B136-33/PACS2/PA7 |
| nosR_R | GAGCAACTCCAGGGTCCAG |  |  |
| algF_F | TCGAAGCTGACCCTGAAGAC | 143 | PAO1/LESB58/DK2/PA14/RP73/B136-33/PACS2 |
| algF_R | TTCAGGTCGCTGACCTTCTT |  |  |
| algA_F | CAAGCAGTACCCCAAGCAGT | 121 | PAO1/LESB58/DK2/PA14/RP73/B136-33/PACS2/PA7 |
| algA_R | GTGCTCCTTGTTGCACACC |  |  |
| glpD_F | GTGTTCCTTTGCGAACAGC | 105 | PAO1/LESB58/DK2/PA14/RP73/B136-33/PACS2/PA7 |
| glpD_R | CAGGCGGAATTCGTAGTGTT |  |  |
| lasB_F | GTCATCGACGCCAAGACC | 97 | PAO1/LESB58/DK2/PA14/RP73/B136-33/PACS2/PA7 |
| lasB_R | ACTTGCCGATCTTCTGGTTG |  |  |
| PA4171_F | CCTGGTCAGGGAATTCAGC | 124 | PAO1/LESB58/RP73/B136-33/PACS2 |
| PA4171_R | GATACGCACGCTGGAATAGG |  |  |
| pqsL_F | CGGCTATTTCATCCTCATGC | 100 | PAO1/LESB58/DK2/PA14/RP73/B136-33/PACS2 |
| pqsL_R | GATGCGGGTCTCGAACAG |  |  |
| phzM_F | CGGCGAAGACTTCTACAGCTA | 142 | PAO1/LESB58/DK2/PA14/RP73/B136-33/PACS2 |
| phzM_R | ACCGACGTCGACGAAGCTA |  |  |
| mreB_F | GGCTCGATGGTCGTAGACA | 128 | PAO1/LESB58/DK2/PA14/RP73/B136-33/PACS2 |
| mreB_R | ACGTAGGTGACGATGGCTTC |  |  |
| mexC_F | CGATCTATGCGGATTTCACC | 125 | PAO1/LESB58/DK2/PA14/RP73/B136-33/PACS2 |
| mexC_R | GTAGGGCGTCCCTTCGAC |  |  |
| nfxB_F | GGCAGTCCTACCTGGAAGC | 118 | PAO1/LESB58/DK2/PA14/RP73/B136-33/PACS2 |
| nfxB_R | CCGTAGACCAGGGTGATGAA |  |  |
| pilQ_F | ACCTGGAGAAACTCGACGTG | 107 | PAO1/LESB58/DK2/PA14/RP73/B136-33/PACS2/PA7 |
| pilQ_R | CGGCTGCTCGATGGTATAG |  |  |
| PA5481_F | GAACCTGGTGGAAGACGAGA | 103 | PAO1/LESB58/DK2/PA14/RP73/B136-33/PACS2/PA7 |
| PA5481_R | CAGATACTTGTCGCCCTGGT |  |  |
| betI_F | ACGGCATCATCAGCCACTAC | 125 | PAO1/LESB58/DK2/PA14/RP73/B136-33/PACS2/PA7 |
| betI_R | CGGACTGTCGTCGTAGAGC |  |  |

^a^No mismatches were found when the corresponding primers were aligned to the indicated reference genomes. ^*^The *PA1922* gene of *P. aeruginosa* RP73 contains a 262 bp deletion, causing a frameshift and preliminary stop codon, thereby truncating the predicted amino acid sequence from 653 to 194 amino acids.
